# Gata3 restrains B cell proliferation and cooperates with p18^INK4c^ to repress B cell lymphomagenesis

**DOI:** 10.18632/oncotarget.11746

**Published:** 2016-08-31

**Authors:** Shiqin Liu, Ho Lam Chan, Feng Bai, Jinshan Ma, Alexandria Scott, David J. Robbins, Anthony J. Capobianco, Ping Zhu, Xin-Hai Pei

**Affiliations:** ^1^ Department of Hematology, Peking University First Hospital, Beijing, 100034, China; ^2^ Molecular Oncology Program, Division of Surgical Oncology, Department of Surgery, Miller School of Medicine, University of Miami, FL 33136, Miami; ^3^ Xinjiang Uigur Autonomous Region People's Hospital, Xinjiang, 830001, China; ^4^ The Sheila and David Fuente Graduate Program in Cancer Biology, Miller School of Medicine, University of Miami, FL 33136, Miami; ^5^ Sylvester Cancer Center, Miller School of Medicine, University of Miami, FL 33136, Miami

**Keywords:** Gata3, p18^INK4c^, B cell, lymphoma

## Abstract

GATA3, a lineage specifier, controls lymphoid cell differentiation and its function in T cell commitment and development has been extensively studied. GATA3 promotes T cell specification by repressing B cell potential in pro T cells and decreased GATA3 expression is essential for early B cell commitment. Inherited genetic variation in *GATA3* has been associated with lymphoma susceptibility. However, it remains elusive how the loss of function of *GATA*3 promotes B cell development and induces B cell lymphomas. In this study, we found that haploid loss of *Gata3* by heterozygous germline deletion increased B cell populations in the bone marrow (BM) and spleen, and decreased CD4 T cell populations in the thymus, confirming that *Gata3* promotes T and suppresses B cell development. We discovered that haploid loss of *Gata3* reduced thymocyte proliferation with induction of p18^Ink4c^ (p18), an inhibitor of CDK4 and CDK6, but enhanced B cell proliferation in the BM and spleen independent of p18. Loss of *p18* partially restored Gata3 deficient thymocyte proliferation, but further stimulated Gata3 deficient B cell proliferation in the BM and spleen. Furthermore, we discovered that haploid loss of *Gata3* in p18 deficient mice led to the development of B cell lymphomas that were capable of rapidly regenerating tumors when transplanted into immunocompromised mice. These results indicate that Gata3 deficiency promotes B cell differentiation and proliferation, and cooperates with p18 loss to induce B cell lymphomas. This study, for the first time, reveals that Gata3 is a tumor suppressor specifically in B cell lymphomagenesis.

## INTRODUCTION

Altered cell differentiation has long been observed during tumorigenesis and poor differentiation is strongly linked to worse prognosis. The molecular mechanism of how altered differentiation is linked to tumorigenesis is poorly understood. Lymphomas are the most common hematological malignancy and account for 5.3% of all cancers in the United States [1]. Depending on the cell type affected, lymphomas can be divided into two major categories: T and B cell lymphomas, the latter accounting for more than 80% of cases [2]. However, the etiology of lymphomagenesis remains largely unknown.

GATA3, a lineage specifier, is critical in regulating self-renewal of hematopoietic stem cells [3–5], and controlling lymphoid cell differentiation [6, 7]. The function of GATA3 in T cell commitment and development has been extensively studied [6–9]. More recently, it has been reported that GATA3 promotes T cell specification by repressing B cell potential in pro-T cells [10, 11], and decreased GATA3 expression is essential for early B cell commitment [12]. Clinical studies revealed that somatic mutations in *GATA3* were frequently detected in early T cell precursor acute lymphoblastic leukemia [13] and that inherited genetic variation in *GATA3* is associated with susceptibility to developing lymphoma and acute lymphoblastic leukemia [14, 15], suggesting that GATA3 may play an important role in suppressing lymphoid malignancies.

GATA3 is expressed in 33–45% of peripheral T cell lymphomas and a subset of T cell lymphomas that correlated with poor survival was found to have increased GATA3 expression [16, 17]. In transgenic mice, forced expression of *GATA3* during T cell development induced T cell lymphomas [18]. These findings suggest that GATA3 functions as a tumor-promoting factor in T cells. However, little is known about the role of GATA3 in B cell tumorigenesis.

In addition to cell differentiation, GATA3 also regulates cell proliferation. Notably, two independent groups demonstrated that loss of Gata3 impairs T cell proliferation [3, 19]. Additionally, loss of Gata3 results in impaired cell cycle entry and proliferation of hematopoietic stem cells (HSCs) [5], although a discrepant report that deletion of *Gata3* enhances self-renewal of HSCs without affecting the cell cycle has also been observed [4]. We, and others, found that GATA3 promotes the proliferation of mammary luminal epithelial cells *in vivo* [20] and T cells *in vitro* [19] by suppressing p18^Ink4c^ (p18) expression.

p18 is a member of the INK4 family that inhibits CDK4 and CDK6, whose activation by mitogen-induced D-type cyclins leads to phosphorylation and functional inactivation of RB, p107, and p130 [21, 22]. Deletion or reduced expression of p18 has been observed in different types of human cancers [22, 23]. Expression of p18 is absent in nearly half of Hodgkin lymphoma cases and correlates with shorter survival compared to patients with p18 positive tumors [24]. Moreover, homozygous deletion of *p18* is frequently detected in B cell lymphomas [25, 26] and its deletion in mice promotes the development of various tumors, including medulloblastoma, glioblastoma, tumors of neuroendocrine organs, lungs, mammary and prostate [20, 27–32]. Confoundingly, although p18 loss stimulates T and B cell proliferation in response to mitogenic signals, it rarely leads to lymphoma development in mice [33, 34].

Since Gata3 deficiency results in aberrant differentiation of lymphoid cells and impaired T cell proliferation and p18 is a downstream target of GATA3 that represses lymphoid cell proliferation, we hypothesized that p18 loss can rescue impaired T cell proliferation, allowing us to determine the effect of Gata3 deficiency in lymphoid cell development and tumorigenesis. In the present study, we generated a mutant mouse strain with heterozygous germline deletion of *Gata3* to determine how haploid loss of *Gata3* affects lymphoid cell proliferation, differentiation, and tumorigenesis. We demonstrate that Gata3 suppresses B cell proliferation and differentiation. Notably, Gata3 cooperates with p18 to repress B cell lymphomas, suggesting that Gata3 functions as a tumor suppressor in B cells in addition to its role as a tumor promoter in T cells.

## RESULTS

### Haploid loss of *Gata3* enhances B cell populations in the bone marrow and spleen and reduces T cell populations in the thymus

**Figure 1:**
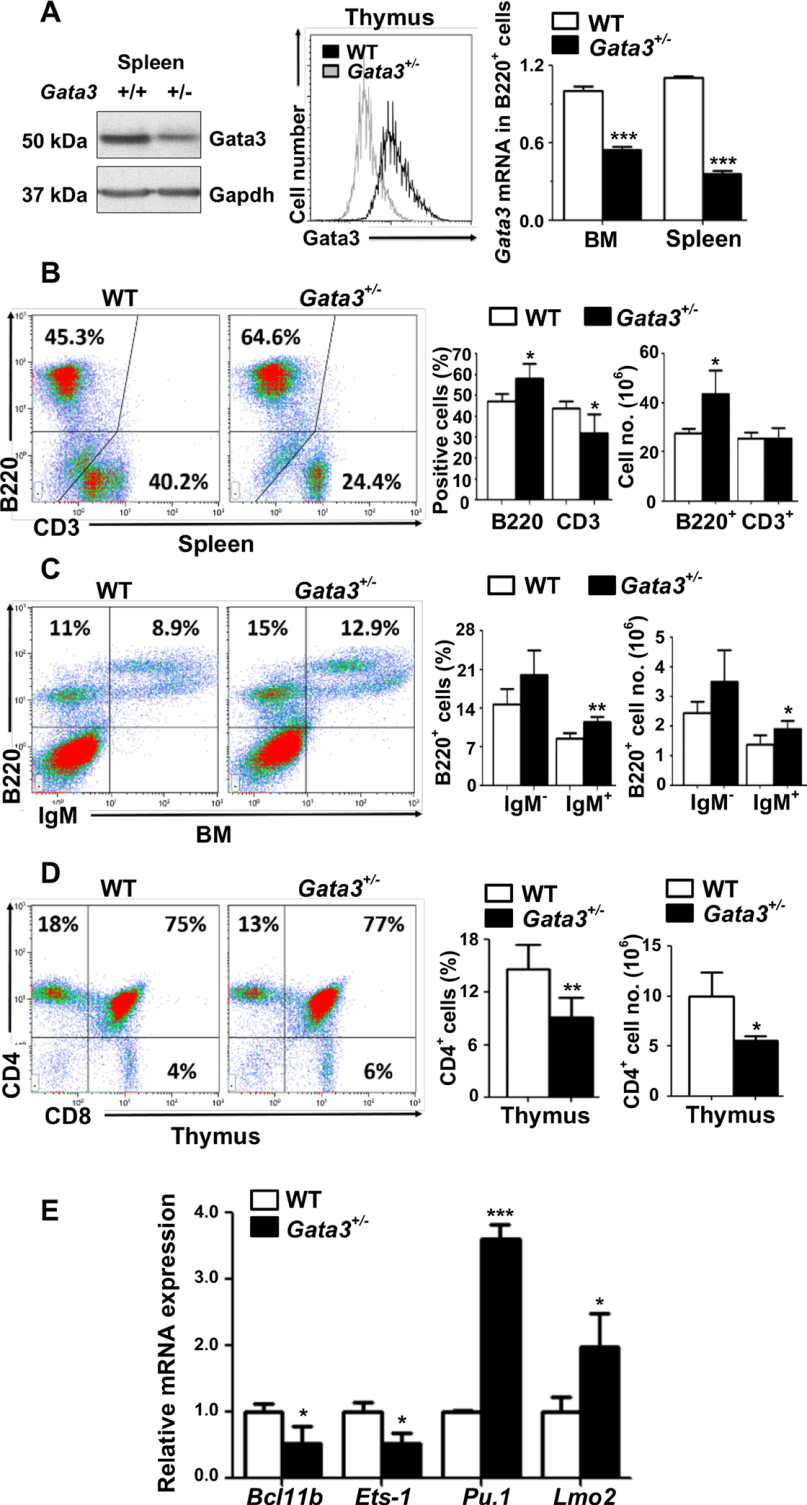
Haploid loss of Gata3 enhances B cell populations in the bone marrow and spleen, but reduces T cell populations in the thymus (**A**) Tissues from the spleen or thymocytes of WT and *Gata3*^+/−^ mice at 2 months of age were analyzed for Gata3 expression by Western blot (left panel) and flow cytometry (middle panel). Sorted B220^+^ B cells from the BM or spleen were analyzed for *Gata3* expression by Q-RT-PCR (right panel). Data represent the mean ± SD from triplicates of 2 mice per genotype. (**B**–**D**). Cells from the spleen, BM and thymus of WT (*n* = 5) and *Gata3*^+/−^ (*n* = 5) mice at 2 months of age were analyzed by flow cytometry. Representative profiles are shown. no., number. (**E**) mRNA levels of the indicated genes in thymocytes from WT and *Gata3*^+/−^ mice at 2 months of age were determined by Q-RT-PCR. Data represent the mean ± SD from triplicates of 3 mice per genotype.

Due to the early embryonic lethality caused by homozygous germline deletion of *Gata3* in mice, the role of *Gata3* in regulation of multiple cell lineages including mammary epithelial cells, hematopoietic stem cells, lymphoid progenitors, and T cells has been investigated using conditional *Gata3* deletion *in vivo* and *ex vivo* [35–37]. Since Gata3 functions in multiple cell lineages, we generated germline *Gata3*^+/−^ mice by crossing *Gata3*^f/+^ mice with BALB/c-CMV-cre mice, a germline “Cre-deleter” strain [38] which enabled us to determine the effects of haploid loss of Gata3 in all cell lineages. We confirmed reduced Gata3 expression in *Gata3*^+/−^ thymocytes, splenocytes and B cells (B220^+^) from the bone marrow (BM) and spleen (Figure 1A and Supplementary Figure S1A). Characterization of *Gata3*^+/−^ mice revealed that there was a significant increase in splenic B220^+^ cells relative to WT counterparts (57.7% ± 6.0% vs. 48% ± 3.2%, *p* < 0.05, Figure 1B). In BM, the B220^+^IgM^+^ (mature B) cell population was significantly increased (11.4% ± 1.1% vs. 8.6% ± 1.0%, *p* < 0.05) and the B220^+^IgM^−^ (immature B) cell population was enhanced (20.0% ± 4.5% vs. 14.7% ± 2.8%, *p* = 0.56) compared to WT littermates (Figure 1C). Though the percentage of the splenic CD3^+^ T cell population was significantly decreased in *Gata3*^+/−^ spleens relative to WT age-matched counterparts (32.0% ± 7.7% vs. 42.8% ± 3.8%, Figure 1B), the absolute numbers of splenic CD3^+^ T cells were comparable between *Gata3*^+/−^ and WT spleens (25.42 × 10^6^ vs. 25.41 × 10^6^, Figure 1B and Supplementary Figure S3B), suggesting that the percentage decrease of splenic CD3^+^ T cells in *Gata3*^+/−^ spleens are relative to the increase of splenic B cells. Notably, the percentage and absolute number of CD4^+^ T cells were significantly decreased in *Gata3*^+/−^ thymi relative to WT counterparts (9.1% ± 2.2% vs. 14.6% ± 2.8%, and 5.58 × 10^6^ vs. 9.98 × 10^6^, Figure 1D and Supplementary Figure S3A), consistent with previous reports [8, 39]. We determined the expression of a few transcription factors regulated by Gata3, *Pu.1* and *Lmo2* which are critical for B cell development, and *Ets- 1* and *Bcl11b* which are important in T cell development [10, 11, 40]. We found that the expression of *Ets-1* and *Bcl11b* was significantly decreased but *Pu.1* and *Lmo2* were significantly increased in *Gata3*^+/−^ thymocytes when compared to WT counterparts (Figure 1E). These results suggest that haploid loss of *Gata3* increases B cell populations in the BM and spleen, and decreases CD4^+^ thymocyte populations, consistent with previous findings showing that Gata3 promotes T and suppresses B cell differentiation [10–12].

### Haploid loss of *Gata3* reduces thymocyte proliferation with induction of p18, but enhances B cell proliferation in the bone marrow and spleen

**Figure 2:**
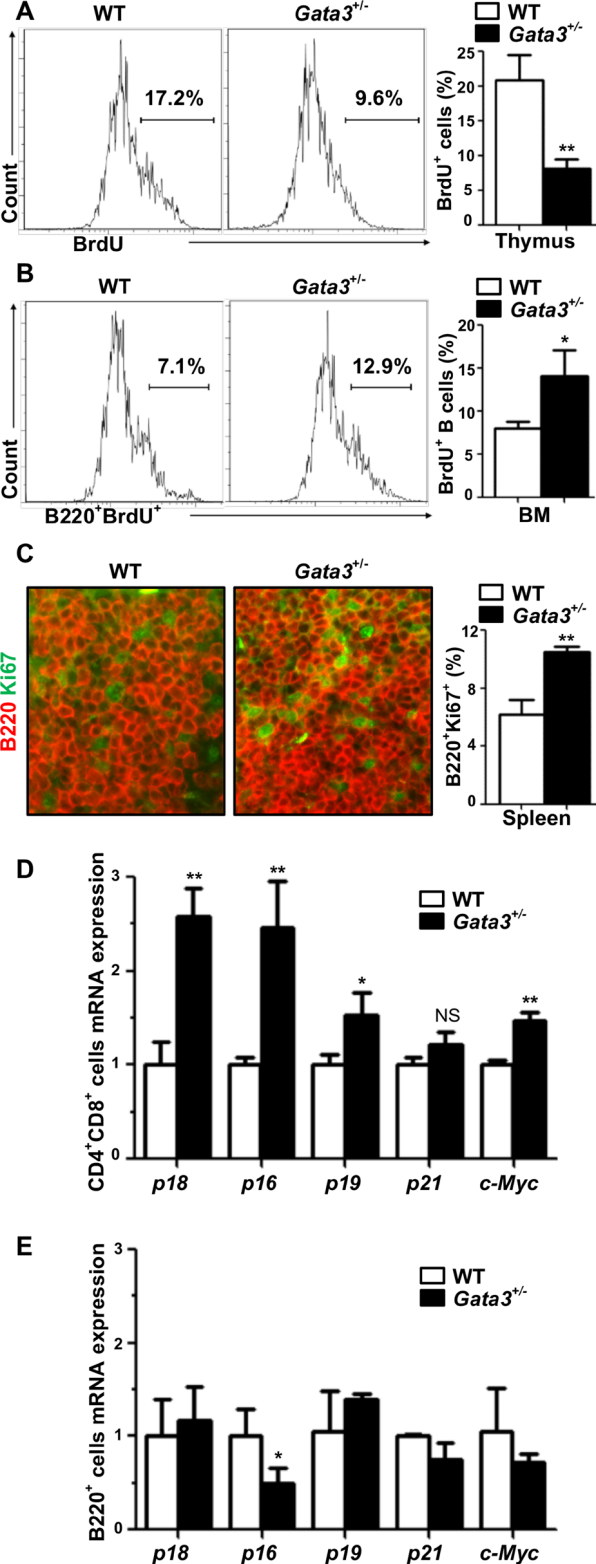
Haploid loss of Gata3 reduces thymocyte proliferation, but stimulates B cell proliferation in the bone marrow and spleen (**A**, **B**) BrdU incorporation in thymocytes and B220^+^ BM cells from WT and *Gata3*^+/−^ mice at 2 months of age were analyzed by flow cytometry. Results represent the mean ± SD of 3 animals per group. (**C**) Immunofluorescence staining of Ki67 (green, nuclear staining) and B220 (red, membrane staining) in spleens from WT and *Gata3*^+/−^ mice. The percentages of Ki67^+^ cells were calculated from B220^+^ cells and quantitated in five randomly selected fields in splenic sections of WT and *Gata3*^+/−^ mice, and the results represent the mean ± SD of three animals per group. (**D**, **E**) Q-RT-PCR analysis for FACS sorted CD4^+^ CD8^+^ cells from the thymus and B220^+^ cells from BM at 2 months of age. Data represent the mean ± SD from triplicates of 2 mice per genotype.

There is growing evidence suggesting that *GATA3* controls proliferation of stem cells and progenitors [3–5, 20] in addition to its role in cell differentiation. We hypothesized that *Gata3* regulates both T and B cell proliferation. To test this hypothesis, we analyzed BrdU incorporation and Ki67 staining in thymocytes and in B cells isolated from the BM and spleen. Consistent with previous findings [3, 19], we detected a significant decrease of thymocyte proliferation by haploid loss of *Gata3*, as evidenced by the reduced BrdU incorporation and less Ki67^+^ cells in *Gata3*^+/−^ thymocytes in comparison with WT counterparts (8.1% ± 1.3% vs. 20.9% ± 3.6% for BrdU incorporation and 14.4% ± 1.9% vs. 24.8% ± 3.7% for Ki67 positivity, Figure 2A and Supplementary Figure S4). Surprisingly, we observed a significant increase of BrdU^+^B220^+^ BM cells and Ki67^+^B220^+^ splenocytes in *Gata3*^+/−^ mice relative to WT counterparts (14.1% ± 2.9% vs. 8.0% ± 0.8% for BrdU^+^B220^+^ cells and 10.5% ± 0.35% vs. 6.2% ± 1.0% for Ki67^+^B220^+^ cells, Figure 2B, 2C), suggesting that haploid loss of Gata3 enhances B cell proliferation in the spleen and BM. These results also indicate that the increased B cell populations in *Gata3*^+/−^ BM and spleen partially attributed to the enhanced B cell proliferation.

It has been reported that depletion of Gata3 impairs T cell proliferation through upregulation of cell cycle inhibitor p18 [19] or down-regulation of c-Myc [3], which both play an important role in lymphoid cell proliferation [33, 34, 41–43]. Since the cell cycle inhibitors p16^Ink4a^ (p16), p19^Ink4d^ (p19), and p21^Cip1^ (p21) also play critical roles in preventing proliferation of cells [22], we then determined the expression of p18 and c-Myc, as well as p16, p19, and p21, in lymphoid cells. We found that the expression of p18, p16, p19, and c-Myc were significantly increased in *Gata3*^+/−^ CD4^+^CD8^+^ thymocytes (Figure 2D). Except in the case of p16, the expression of these genes in *Gata3*^+/−^ B220^+^ BM cells were not significantly changed compared to WT (Figure 2E). Interestingly, *p16* mRNA in *Gata3*^+/−^ B220^+^ BM was significantly reduced relative to WT cells (Figure 2E), which may contribute to the increased B cell proliferation in *Gata3*^+/−^ mice. We previously demonstrated that p18 is a downstream target of GATA3 and restrains mammary luminal progenitor cell proliferation and tumorigenesis [20]. Recently, it has also been confirmed that GATA3 regulates T cell proliferation through repression of p18 *in vitro* [19]. These findings prompted us to hypothesize that p18 loss may rescue the T cell proliferative defects as a result of Gata3 deficiency, allowing us to investigate the long term effects of Gata3 deficiency in lymphoid malignancies. To test this hypothesis, we crossed *p18* null mice with *Gata3*^+/−^ mice and generated *p18*^−/−^;*Gata3*^+/−^ mice.

### Loss of p18 partially restores thymocyte proliferation but further stimulates B cell proliferation induced by Gata3 deficiency

**Figure 3:**
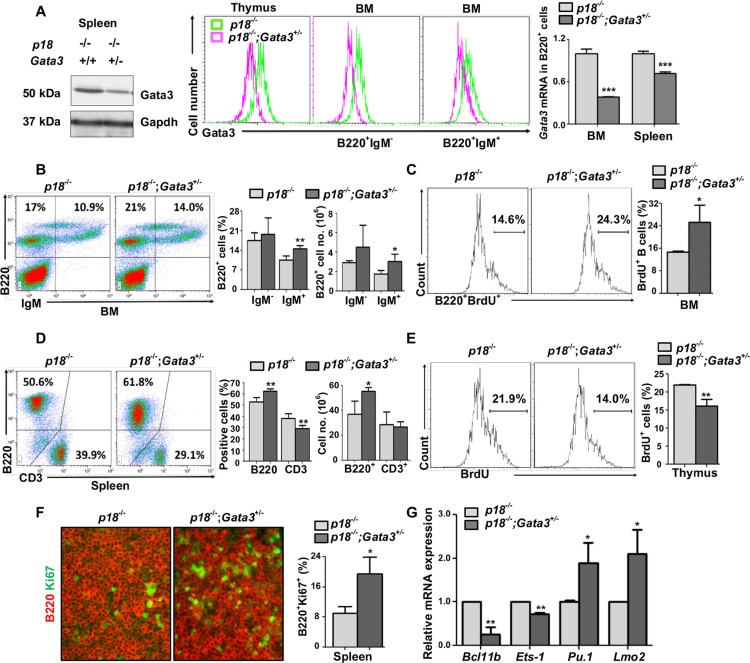
Loss of p18 partially restores Gata3 deficient thymocyte proliferation, and haploid loss of Gata3 in a p18 null background promotes B cell proliferation and differentiation (**A**). Splenic tissue, thymocytes, or sorted BM B cells (B220^+^IgM^−^and B220^+^IgM^+^) from *p18*^−/−^ and *p18*^−/−^;*Gata3*^+/−^ mice at 2 months of age were analyzed for Gata3 expression by Western blot (left panel) and flow cytometry (middle panel). Sorted B220^+^ B cells from the spleen and BM were analyzed for *Gata3* expression by Q-RT-PCR (right panel). Data represent the mean ± SD from triplicates of 2 mice per genotype. (**B**, **D**) Cells from the BM (B) and spleen (D) of *p18*^−/−^ and *p18*^−/−^;*Gata3*^+/−^ mice at 2 months of age were analyzed. Results represent the mean ± SD of 5 animals per group. (**C**, **E**) BrdU incorporation in B220^+^ BM cells (C) and thymocytes (E) from *p18*^−/−^ and *p18*^−/−^;*Gata3*^+/−^ mice at 2 months of age were analyzed by flow cytometry. Results represent the mean ± SD of 3 animals per group. (**F**) Immunofluorescence staining of Ki67 (green, nuclear staining) and B220 (red, membrane staining) in the spleen from *p18*^−/−^ and *p18*^−/−^;*Gata3*^+/−^ mice. The percentagest of Ki67^+^ cells were calculated from B220^+^ cells and quantitated in five randomly selected fields in splenic sections of WT and *Gata3*^+/−^ mice, and the results represent the mean ± SD of three animals per group. (**G**) mRNA levels of the indicated genes in thymocytes from *p18*^−/−^ and *p18*^−/−^;*Gata3*^+/−^ mice at 2 months of age were determined by Q-RT-PCR. Data represent the mean ± SD from triplicates of 3 mice per genotype.

It has been reported that p18 deficient T and B lymphocytes display a hyperproliferative response to mitogenic signals [33]. We performed FACS and found that CD4/CD8 and B220/IgM profiles in the cells from *p18*^−/−^ thymus, spleen, and BM were comparable to the cells from WT counterparts (Figure 3D vs. Figure 1B; Figure 3B vs. Figure 1C, Supplementary Table S1, and data not shown), indicating that loss of *p18* does not affect T and B cell development, consistent with previous findings [33]. We then determined cell proliferation and found that there were slightly more BrdU^+^ and Ki67^+^ thymocytes in *p18*^−/−^ mice than in WT counterparts at two months of age (22.0% ± 0.1% vs. 20.9% ± 3.6% for BrdU^+^ thymocytes, *p* > 0.05, 30.5% ± 3.6% vs. 24.8% ± 3.8% for Ki67^+^ thymocytes, *p* > 0.05, Figure 3E and Figure 2A, Supplementary Table S2, and Supplementary Figure S4). However, BrdU^+^ and Ki67^+^ B cells in *p18*^−/−^ BM and spleen were significantly increased relative to WT counterparts (14.8% ± 0.3% vs. 8.0% ± 0.8% for BrdU^+^B220^+^ BM cells, *p* < 0.05, Figure 2B and 3C, 9.4% ± 1.2% vs. 6.2% ± 1.0% for Ki67^+^B220^+^ splenocytes, *p* < 0.05, Figures 2C and 3F). These results suggest that loss of p18 stimulates B cell proliferation in mice under homeostatic conditions.

*p18*^−/−^;*Gata3*^+/−^ thymocytes, splenocytes, B220^+^IgM^−^ immature B and B220^+^IgM^+^ mature B cells in BM, expressed less *Gata3* mRNA and protein than *p18*^−/−^ cells (Figure 3A and Supplementary Figure S1B). Consistently, we also observed that in the BM, *Gata3*^+/−^ B220^+^IgM^−^ and B220^+^IgM^−^ cells expressed less Gata3 than WT counterparts (Supplementary Figure S1C). We determined lymphocyte distribution in 2-month-old mice and found that *p18*^−/−^;*Gata3*^+/−^ spleen and BM displayed significantly more B220^+^ and B220^+^IgM^+^ cells than *p18*^−/−^ counterparts, respectively (Figure 3B, 3D, and Supplementary Table S1). Although the percentages of CD3^+^ splenocytes and CD4^+^ thymocytes in *p18*^−/−^;*Gata3*^+/−^ mice were significantly reduced when compared with *p18*^−/−^ mice (29.1% ± 2.9% vs. 38.1% ± 4.4% for CD3^+^ splenocytes, *p* = 0.005, 8.6% ± 1.3% vs. 11.8% ± 2.3% for CD4^+^ thymocytes, *p* = 0.03, Figure 3D and Supplementary Table S1), the absolute number of these cells in *p18*^−/−^;*Gata3*^+/−^ mice, CD4^+^ thymocytes in particular, was not significantly decreased (Figure 3D and data not shown). In line with the findings derived from *Gata3*^+/−^ thymocytes (Figure 1E), we also detected a significantly increased expression of *Pu.1* and *Lmo2,* as well as significantly decreased expression of *Ets-1* and *Bcl11b* in *p18*^−/−^;*Gata3*^+/−^ thymocytes relative to those in *p18*^−/−^ counterparts (Figure 3G). Together, these results suggest that haploid loss of *Gata3* in *p18* null mice promotes B cell development in the BM and spleen. How Gata3 deficiency impacts T cell development in a p18 null background needs further investigation.

We then determined lymphoid cell proliferation in *p18*^−/−^;*Gata3*^+/−^ mice at young age. We detected significantly more BrdU^+^B220^+^ BM cells and Ki67^+^B220^+^ splenocytes in *p18*^−/−^;*Gata3*^+/−^ mice at two months of age when compared to *p18*^−/−^ counterparts (27.5% ± 5.1% vs. 14.8% ± 0.3% for BrdU^+^B220^+^ cells and 19.5% ± 4.4% vs. 9.4% ± 1.2% for Ki67^+^B220^+^ cells, Figure 3C, 3F and Supplementary Table S2). These data indicate that loss of p18 collaborates with Gata3 deficiency to stimulate B cell proliferation. Importantly, BrdU incorporation in *p18*^−/−^;*Gata3*^+/−^ thymocytes were significantly less than in *p18*^−/−^ cells (16.1% ± 1.9% vs. 22.0% ± 0.1%) and incorporation in both these cells were significantly more than *Gata3*^+/−^ thymocytes (16.1% ± 1.9% and 22.0% ± 0.1% vs. 8.1% ± 1.3%, Figures 3E and Figure 2A). In addition, BrdU^+^ thymocytes in *p18*^−/−^;*Gata3*^+/−^ were not as high as in WT cells (16.1% ± 1.9% vs. 20.9% ± 3.6%, Figure 3E and Figure 2A). To consolidate this finding, we also performed Ki67 staining in the thymus and found that there were significantly less Ki67^+^ thymocytes in *p18*^−/−^;*Gata3*^+/−^ mice than in *p18*^−/−^ mice, but more than in *Gata3*^+/−^ mice (Supplementary Figure S4). These data suggest that loss of p18 partially rescues growth defects in thymocytes caused by Gata3 deficiency. Taken together, these results further suggest that Gata3 promotes thymocyte proliferation partially through the repression of p18, whereas Gata3 collaborates with p18 to inhibit B cell proliferation in the BM and spleen.

### Haploid loss of Gata3 in a p18 deficient background leads to development of B cell lymphomas

**Figure 4:**
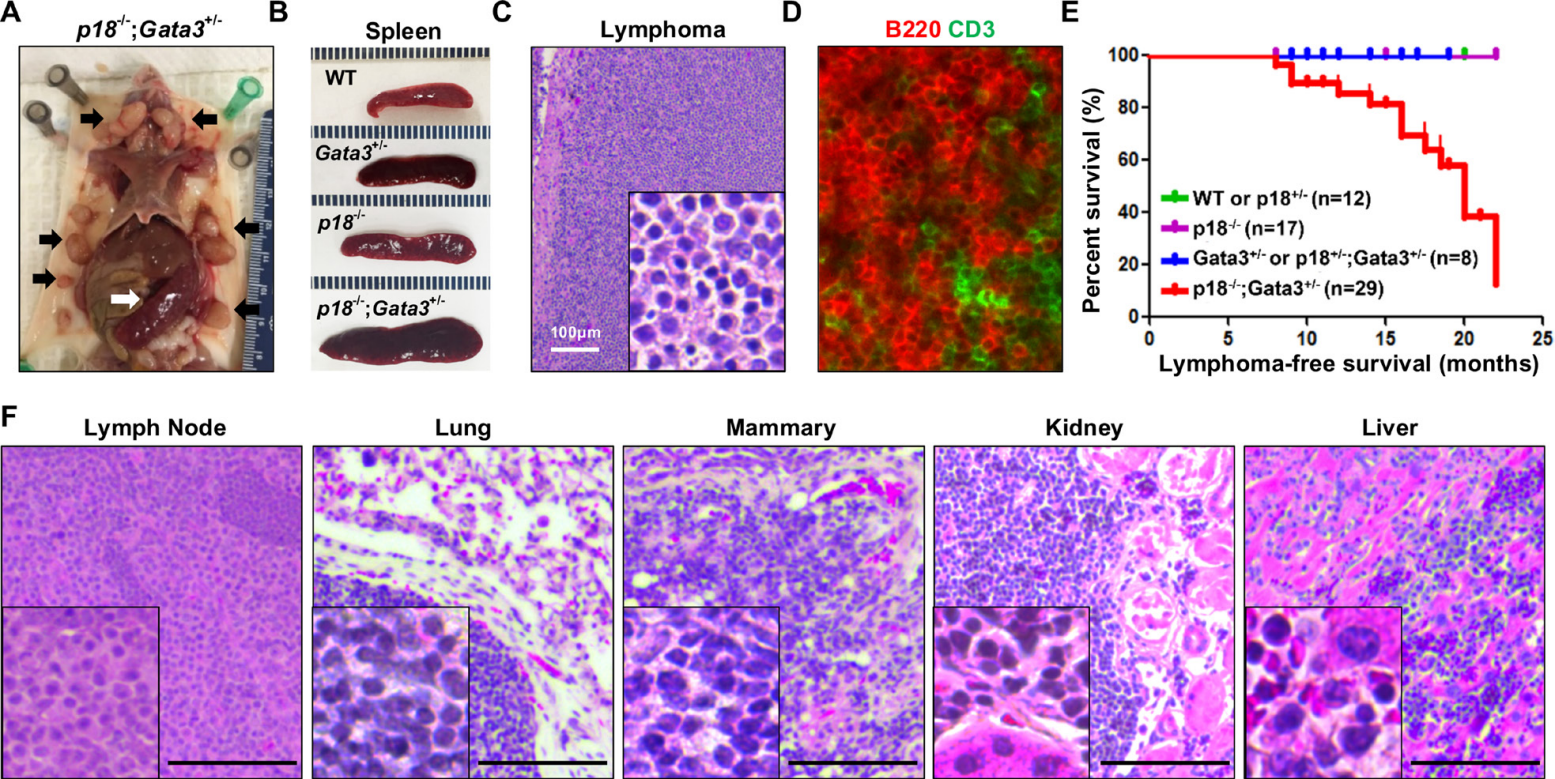
Gata3 deficiency in p18 null mice induces B-cell lymphomas (**A**) Representative lymphomas developed in *p18*^−/−^;*Gata3*^+/−^ mice. Note the multiple enlarged lymph nodes (black arrows) and typical enlarged spleen (white arrow). (**B**) Representative gross appearance of spleens from age-matched (12–14 months of age) WT, *Gata3*^+/−,^ *p18*^−/−^ and *p18*^−/−^;*Gata3*^+/−^ mice. (**C**, **D**) Representative H&E (C) and immunofluorescence staining (D) of primary lymphomas developed in *p18*^−/−^;*Gata3*^+/−^mice. (**E**) Lymphoma-free survival of mice with different genotypes. *p* = 0.018 among four groups by Log-rank (Mantel-Cox) Test. (**F**) Representative *p18*^−/−^;*Gata3*^+/−^ lymphomas infiltrated into multiple organs, determined by H&E staining.

**Figure 5:**
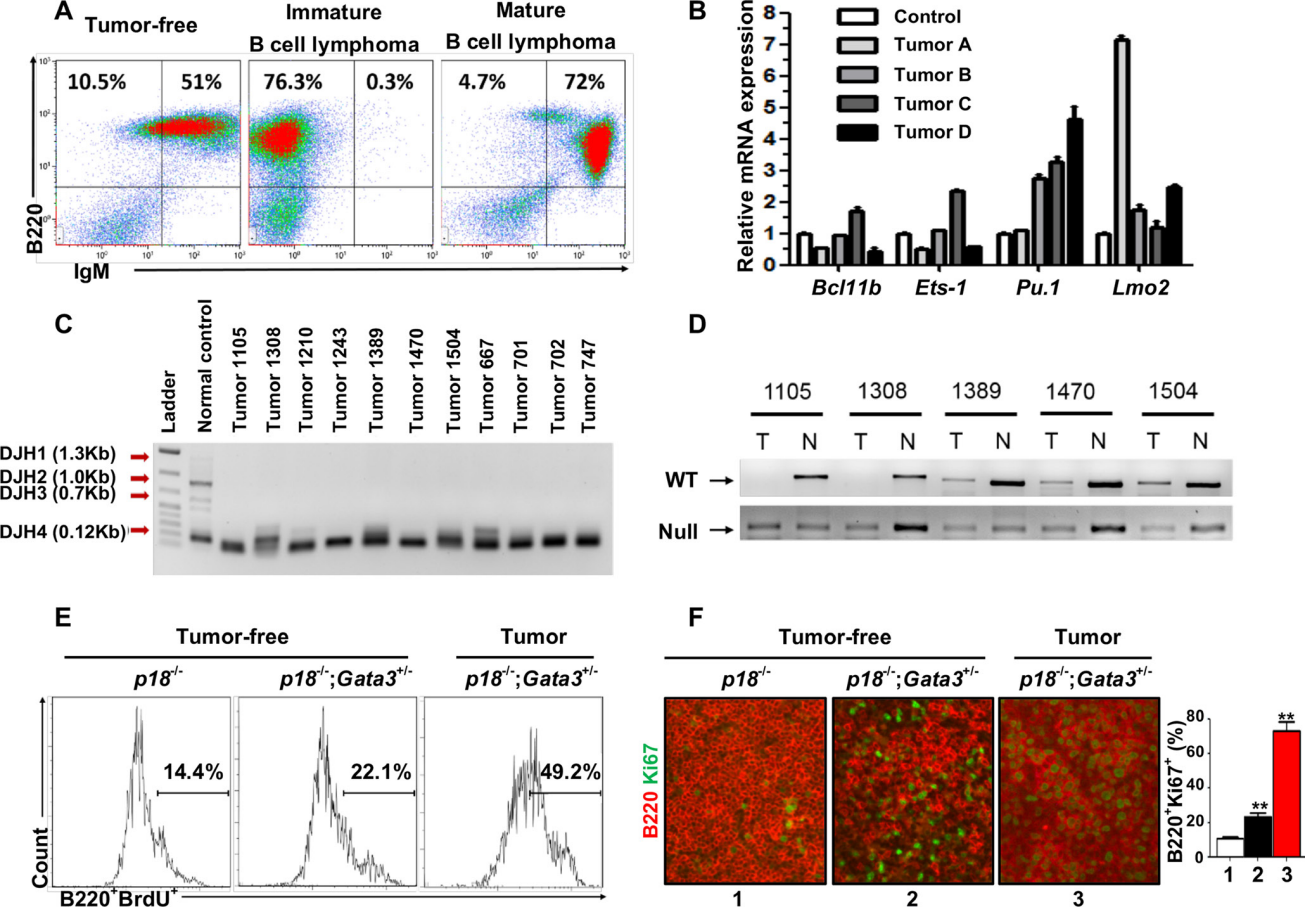
Characterization of lymphomas developed in *p18*^−/−^;*Gata3*^+/−^ mice (**A**) Representative lymphomas from *p18*^−/−^;*Gata3*^+/−^ mice at 14–16 months of age were analyzed by FACS with the indicated antibodies. Age-matched spleens from tumor-free mice of the same genotype were used as control. (**B**) Q-RT-PCR analysis for cells derived from four representative *p18*^−/−^;*Gata3*^+/−^lymphomas. Splenocytes from the age-matched, lymphoma-free mice of the same genotype were used to normalize the expression for each gene. Data represent the mean ± SD from triplicate experiments. (**C**) DJ rearrangement of the heavy chain of immunoglobulin in DNA from *p18*^−/−^;*Gata3*^+/−^ lymphomas was determine by PCR. DNA from a WT spleen with polyclonal B-cell populations was shown as a control. (**D**) Loss of heterozygosity analysis of representative *p18*^−/−^;*Gata3*^+/−^ lymphomas. DNA isolated from microdissected lymphomas or ear tissues from the same mice were analyzed by PCR. (**E**) Representative BrdU incorporation in cells from a *p18*^−/−^;*Gata3*^+/−^ lymphoma (Lane 3) and splenocytes from the age-matched (14–6 months), *p18*^−/−^ (Lane 1) and *p18*^−/−^;*Gata3*^+/−^ (Lane 2) lymphoma-free mice. (**F**) Immunofluorescence staining of Ki67 (green, nuclear staining) and B220 (red, membrane staining) in spleens from *p18*^−/−^ (Lane 1) and *p18*^−/−^;*Gata3*^+/−^ (Lane 2) lymphoma-free mice and a *p18*^−/−^;*Gata3*^+/−^ lymphoma (Lane 3). The percentages of Ki67^+^ cells were calculated from B220^+^ cells and quantitated in five randomly selected fields in splenic sections, and the results represent the mean ± SD of three animals per group.

We followed *p18* and *Gata3* single and combined mutant mice to older ages and found that the spleens were dramatically enlarged in *p18*^−/−^ and *p18*^−/−^;*Gata3*^+/−^mice, respectively, starting at 8 months of age when compared with WT and *Gata3*^+/−^ counterparts (Figure 4A, 4B). The majority of *p18*^−/−^;*Gata3*^+/−^ mice displayed various degrees of lymphadenopathy whereas no enlargement of the thymus, spleen nor lymph nodes were detected in WT and *Gata3*^+/−^ mice at a similar age. Histological analysis revealed that some *p18*^−/−^;*Gata3*^+/−^ mice displayed typical lymphoma pathology, such as effacement of normal architecture and uniform cell morphology (Figure 4C, 4F). We performed immunohistochemistry (IHC) and immunofluorescence analysis for *p18*^−/−^; *Gata3*^+/−^ lymphomas and found that the majority of lymphoma cells were B220^+^ and a small portion of the cells were CD3^+^ (Figure 4D). *p18*^−/−^;*Gata3*^+/−^ spleens and lymph nodes with lymphomas exhibited aberrant B220^+^IgM^−^ or B220^+^IgM^+^ cell populations relative to age-matched tumor-free counterparts of the same genotype (Figure 5A). These results suggest that *p18*^−/−^;*Gata3*^+/−^ lymphomas were either B or pre-B cell lymphomas.

We determined the expression of transcription factors related with T and B cell differentiation in four representative *p18*^−/−^;*Gata3*^+/−^ lymphomas. We found that all tumors expressed high levels of *Pu.1* and *Lmo2* relative to lymphoma-free splenocytes from age-matched tumor-free mice with the same genotype (Figure 5B). Notably, except for one lymphoma expressing slightly increased *Ets-1* and *Bcl11b* (Tumor C in Figure 5B), all the remaining lymphomas expressed low or unchanged *Ets-1* and *Bcl11b*. These data confirm that Gata3 also inhibits B cell differentiation during lymphoma development.

After monitoring a cohort of 29 *p18*^−/−^;*Gata3*^+/−^, 17 *p18*^−/−^, 8 *Gata3*^+/−^ or *p18*^+/−^;*Gata3*^+/−^ and 12 WT or *p18*^+/−^ mice for 8–22 months, we found that 46% (*n* = 11) and 50% (*n* = 18) of *p18*^−/−^;*Gata3*^+/−^ mice developed spontaneous lymphomas at 8–14 and 14–22 months of age, respectively, whereas no WT, *Gata3*^+/−^ or *p18*^−/−^ mice formed lymphomas at similar ages (Figure 4 and Table 1). About 1/3 (5 of 14) *p18*^−/−^;*Gata3*^+/−^ lymphomas infiltrated into non-lymphoid organs including the liver, kidney, or lung (Figure 4F). Interestingly, 4 of 14 *p18*^−/−^;*Gata3*^+/−^ mice with lymphomas also developed mammary tumors. The median lymphoma-free survival time was 20 months in *p18*^−/−^;*Gata3*^+/−^ mice and the earliest lymphomas were detected at 8 months of age (Figure 4E). These results indicate that *Gata3* deficiency collaborates with *p18* loss to induce lymphomagenesis, though the loss of either gene alone rarely leads to lymphoma.

**Table 1:** Spontaneous lymphoma development in *p18*^−/−^ *Gata3*^+/−^ mice

Genotype	WT or *p18*^+/−^^a^		*p18*^−/−^		*Gata3*^+/−^ or *p18*^+/−^;*Gata3*^+/−^^b^		*p18*^−/−^;*Gata3*^+/−^	
Age(Months)	8–14	14–22	8–14	14–22	8–14	14–22	8–14	14–22
B-cell Lymphoma	0/8	0/4	0/7	0/10	0/5	0/3	5/11 (46%)	9/18 (50%)

a This group includes 7 *p18*^+/−^ mice.

b This group includes 6 *p18*^+/−^;*Gata3*^+/−^ mice.

To determine the presence of clonal B cells in lymphomas, somatic recombination at the immunoglobulin heavy chain (*IgH)* locus in lymphomas were analyzed by PCR as previously described [44]. We found that all lymphomas exhibited the same clonal rearrangement (DHJ4 0.12kb), indicating that the lymphomas developed from *p18*^−/−^;*Gata3*^+/−^ mice were clonal (Figure 5C). We performed loss of heterozygosity (LOH) analysis for *p18*^−/−^;*Gata3*^+/−^ lymphomas and found that the remaining WT allele of *Gata3* is absent in at least 2 out of 5 tumors examined (Figure 5D), further supporting the role of Gata3 in suppressing B cell lymphomas. Due to the very low level of GATA3 expression in B cells that is undetectable by western blot [3] and IHC (Pei unpublished data), we were unable to determine whether Gata3 expression is further reduced in tumor cells of *p18*^−/−^;*Gata3*^+/−^lymphomas with LOH relative to lymphoma-free B cells.

We then performed FACS and IHC and found that in lymphoma-free mice at older ages (14–16 months), B220^+^ cells from *p18*^−/−^;*Gata3*^+/−^ spleens incorporated more BrdU and had a higher percentage of Ki67^+^ cells (Figure 5E, 5F) than those from age-matched *p18*^−/−^ cells. Further, the percentages of BrdU^+^ or Ki67^+^ B cells from *p18*^−/−^; *Gata3*^+/−^ lymphomas were significantly more than cells from lymphoma-free spleens of the same genotype (Figure 5E, 5F). These data, in addition to the results described above, indicate that Gata3 deficiency cooperates with p18 loss to stimulate B cell proliferation from a young age and to maintain an expanded B cell population throughout life, eventually leading to B cell lymphomas.

### *p18;Gata3* double mutant lymphoma cells rapidly form lymphoma in recipient mice

**Figure 6:**
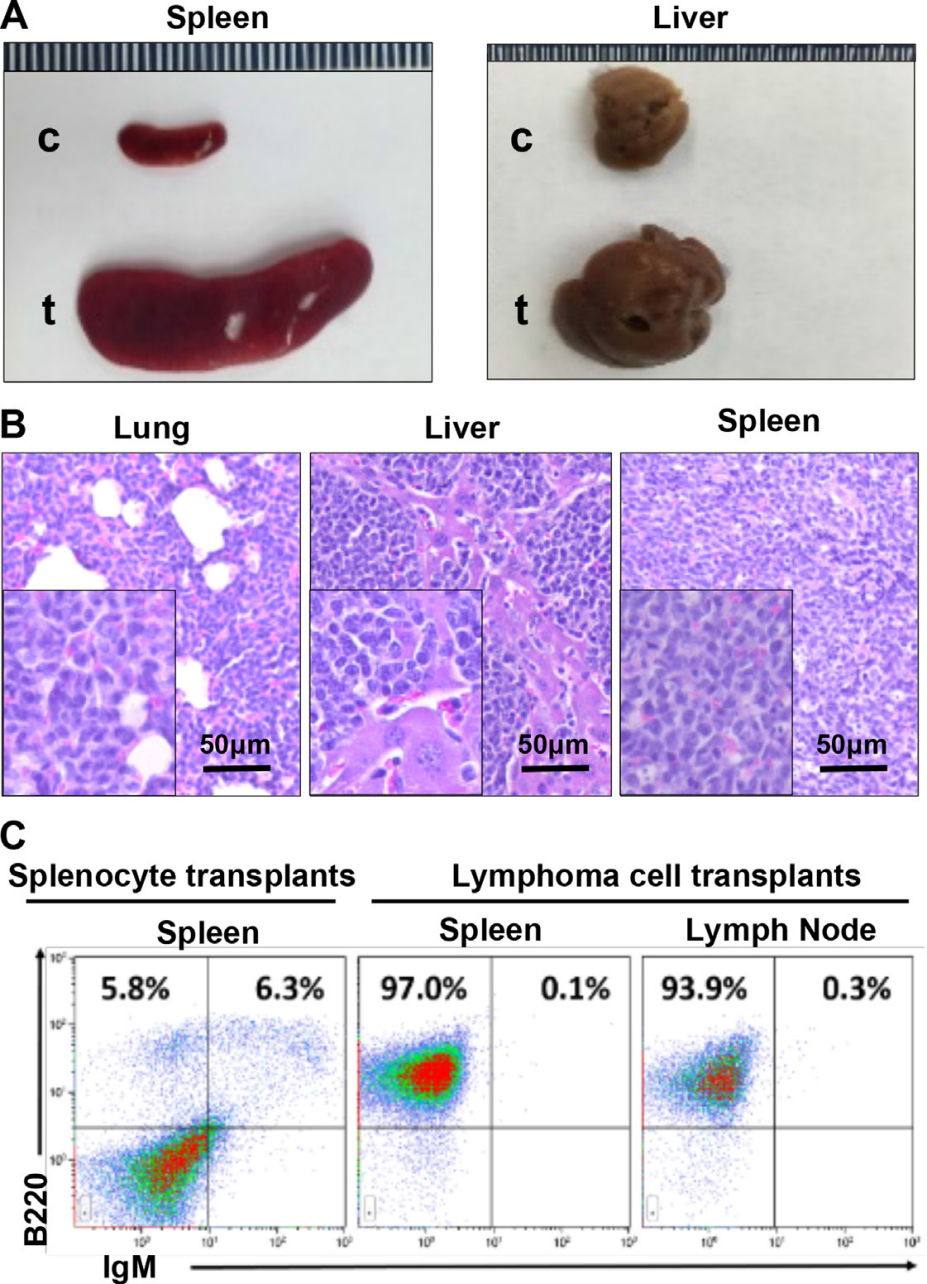
*p18*^−/−^;*Gata3*^+/−^ lymphoma cells are transplantable (**A**) Representative gross appearance of spleens and livers from recipient mice transplanted by *p18*^−/−^;*Gata3*^+/−^ lymphoma cells (t) and asymptomatic splenocytes (c). (**B**) Representative H&E staining for lung, liver, and spleen in mice that received lymphoma cell transplants. Note massiave lymphoma cells infiltrating into these organs. (**C**) Representative FACS analysis for the cells isolated from receipient mouse spleens and lymph nodes. Splenocytes from the mice that received asymptomatic splenocyte transplants were used and analyzed as control.

We then transplanted two million cells isolated from a *p18*^−/−^;*Gata3*^+/−^ splenic lymphoma (immature B-cell lymphoma) or an asymptomatic spleen into each female NSG mouse (*n* = 3 for each group) by tail vein injection. After one month, all mice that received lymphoma cell transplants exhibited clinical signs of illness such as hunched posture, loss of appetite, ruffled fur and dyspnea whereas no symptoms were observed in mice that received control splenocytes. Necropsy and histological analysis revealed that mice that received lymphoma cell transplants displayed marked lymphadenopathy and enlarged spleens, with tumor cells infiltrated into multiple non-lymphoid organs including liver and lung (Figure 6A, 6B). Flow cytometry analysis showed that more than 90% of cells from spleen, lymph node, and BM of the lymphoma cell transplants exhibited aberrant B220^+^ IgM^−^ cell populations (Figure 6C), which were comparable with the populations in the donor lymphoma (Figure 5A, middle panel). No mice received control splenocytes displayed similar flow cytometry profiles in splenocytes (Figure 6C). Together, these results demonstrate that *p18*^−/−^;*Gata3*^+/−^ lymphoma cells are transplantable and highly tumorigenic into secondary immunodeficient mice.

## DISCUSSION

In this study, we found that heterozygous germline deletion of *Gata3* increased the B cell populations in the BM and spleen, but decreased CD4 T cell populations in the thymus, confirming the finding that Gata3 promotes T and suppresses B cell differentiation. Importantly, we discovered that heterozygous germline deletion of *Gata3* reduced thymocyte proliferation with induction of p18 but enhanced B cell proliferation in the BM and spleen independent of p18. Moreover, loss of p18 partially restored Gata3 deficient thymocyte proliferation but further stimulated Gata3 deficient B cell proliferation in the BM and spleen. We found that heterozygous germline deletion of *Gata3* in p18 deficient background led to development of B cell lymphomas. These results indicate that Gata3 deficiency promotes B cell differentiation and proliferation, and cooperates with p18 loss to induce B cell lymphomas. To the best of our knowledge, this is the first genetic evidence showing that loss of function of *Gata3* promotes B cell lymphomagenesis. Our results suggest that Gata3 is a tumor suppressor in B cells as opposed to a tumor promoter in T cells.

Overexpression of GATA3 in T cells induces T cell lymphomas in mice [18]. Two clinical studies showed that GATA3 is aberrantly overexpressed in a subset of T cell lymphomas [16, 17]. Notably, GATA3 is undetectable in the majority of B-cell lymphomas including the nodular lymphocyte predominance type of Hodgkin lymphomas and various B cell non-Hodgkin lymphomas whereas it is aberrantly expressed in some of the Hodgkin and Reed/Sternberg cells (HRS) cells [45–47]. Classical Hodgkin lymphoma (HL) is unique among human lymphomas in the extent to which the lymphoma cells have undergone reprogramming of gene expression and the hallmark of this disease is the presence of HRS cells [48]. Despite their B cell origin, HRS cells have lost expression of many B cell markers and acquired expression of multiple markers that are not found in normal B cells [48]. It has been shown that the deregulated constitutive activity of NF-kB and Notch-1 alters GATA3 expression in HRS cells [46]. Furthermore, GATA3 activity correlates with IL-5 and IL-13 expression in HRS cells, which may contribute to the pathogenesis of HL [46]. Importantly, HRS cells account for only about 1% of cells in the tumor tissue and the majority of cells in classical HL are a mixed immune infiltrate comprising of CD4^+^ cells. A recent study has reported that CD4-associated GATA3 is expressed at significantly low levels in classical HL tissue [49], suggesting that GATA3 deficiency may contribute to HL development through deregulating CD4^+^ cells in the HL microenvironment. Furthermore, it has been identified that inherited genetic variation of *GATA3* is associated with susceptibility to developing HL, though the risk allele is not associated with *GATA3* gene expression [14]. However, whether GATA3 is required for development of HRS and HL remains to be investigated. We demonstrated that haploid loss of *Gata3* in mice enhances B cell differentiation at young age and leads to B cell lymphomas in aged mice in the absence of a cell cycle inhibitor, p18. These results suggest that Gata3 abundance is critical in controlling lymphomagenesis with a lineage specific effect, i.e. high expression of GATA3 causes T cell lymphomas and Gata3 deficiency results in B cell lymphomas.

Genome-wide association studies have revealed that inherited genetic variation in *GATA3* is associated with susceptibility to developing B cell acute lymphoblastic leukemia and the B cell leukemia risk allele is associated with increased GATA3 expression [15, 50]. Though it is unexpected, the increased risk of a B cell leukemia by overexpression of a non-B-cell-specific transcription factor, e.g. GATA3, is not surprising. Wiemels et al., recently discovered that a B-cell leukemia risk allele in the myeloid specific transcription factor *CEBPE* is also associated with increased gene expression [51]. These observations suggest that the increased *GATA3* and *CEBPE* expression associated with B cell leukemia risk alleles may be associated with the lineage confusion, a common feature of leukemogenesis [52].

The function of GATA3 in controlling cell fate and differentiation, lymphoid cell differentiation in particular, has been extensively studied [10–12]. Not until recently has the role of GATA3 in regulating cell proliferation been investigated. In the present study, we demonstrated that heterozygous germline deletion of *Gata3* reduces T cell proliferation with induction of p18, but enhances B cell proliferation, and that loss of p18 partially restores Gata3 deficient T cell proliferation but further stimulates Gata3 deficient B cell proliferation, eventually leading to development of B cell lymphomas. These data indicate that GATA3 regulates cell proliferation in a cell type dependent manner. The molecular mechanisms underlying the role of GATA3 in promoting T cell proliferation through repressing p18 was reported [19] and further supported by our findings in mammary luminal epithelial cells [20]. Though it was shown that depletion of Gata3 in CD8^+^ T cells reduces their proliferation and that c-Myc was identified as a target of Gata3 in promoting CD8^+^ T cell proliferation [3], we failed to detect *c-Myc* mRNA reduction in *Gata3*^+/−^ CD4^+^CD8^+^ T cells (Figure 2D). This may be mainly caused by the different cell populations checked (CD8^+^ vs CD4^+^CD8^+^) and different *Gata3* mutant mice used (Cd4-Cre-*Gata3*^f/f^ vs. germline *Gata3*^+/−^). The mechanisms of GATA3 in suppressing B cell proliferation remains to be determined. It is likely that Gata3 positively regulates p16 in B cells to inhibit their proliferation. Our finding that Gata3 regulates lymphoid cell proliferation in a cell type dependent manner supports a unique role of Gata3 in coordinately controlling lymphoid cell development – Gata3 promotes T cell differentiation and proliferation, but suppresses B cell differentiation and proliferation.

Heterozygous germline deletion of *Gata3* alone does not result in lymphoma development, though aberrantly differentiated B cells proliferate slightly faster than WT B cells at early ages, indicating that haploid loss of *Gata3* is not sufficient to induce lymphomas. Interestingly, *p18*^−/−^;*Gata3*^+/−^ mice develop B, not T, cell lymphomas despite the role of p18 loss in stimulating both T and B cells. These data indicate that Gata3 cooperates with p18 in suppressing B cell proliferation and lymphomagenesis, and that Gata3 promotes T cell proliferation partially through repression of p18. The result that p18 suppresses B cell lymphoma development is supported by the clinical finding that loss of function of p18 is frequently detected in human lymphomas, B cell lymphomas in particular [24–26].

## MATERIALS AND METHODS

### Mice, histopathology and immunofluorescence

The generation and genotyping of *p18*^−/−^ mice have been described previously [33]. BALB/c-Tg (CMV-cre) 1Cgn/J mice were obtained from Jackson Laboratories [38]. *Gata3*^f/f^ mice [37] were kindly provided by Dr. I-Cheng Ho (Harvard Medical School). NOD.*Cg*-*Prkdc*^scid^ Il2rg^tm1Wjl^/SzJ (NSG) mice (6–8 weeks old) were obtained from Jackson Laboratory. The Institutional Animal Care and Use Committee at the University of Miami approved all animal procedures. Histopathology and immunofluorescent staining were performed as described previously [53, 54]. Primary antibodies used were as follows: B220 (eBioscience), CD3 (Abcam) and Ki67 (Abcam). Immunocomplexes were detected using FITC- or rhodamine-conjugated secondary antibodies (Jackson Immunoresearch). For quantification of B220 and Ki67 double positive cells, 5 random fields in at least two cut sections from each spleen were examined and at least 700 B220^+^ cells per field were counted (more than 3500 B220^+^ cells per mouse). The average percentages of Ki67 and B220 double positive cells were calculated from WT, *Gata3*^+/−^, *p18*^−/−^ and *p18*^−/−^;*Gata3*^+/−^ mice and the results represent the mean ± SD of three animals per group. For quantification of Ki67 positive cells in thymus, 5 random fields in at least two cut sections from each thymus were examined and at least 500 DAPI positive cells per field were counted (more than 2500 cells per thymus). The average percentages of Ki67 positive cells were calculated from WT, *Gata3*^+/−^, *p18*^−/−^ and *p18*^−/−^;*Gata3*^+/−^ mice and the results represent the mean ± SD of two animals per group.

### Flow cytometry

Single-cell suspensions from the thymus, spleen, bone marrow (BM), and tumor were obtained. Total cell numbers in each sample was determined by an automatic cell counter (Bio-rad). One million harvested cells were stained with anti-B220-APC, anti-CD3-FITC (Bioscience), anti-IgM-PE, anti-CD4-AF700 and anti-CD8-pacific blue (Biolegend) antibodies. Cells were sorted on a BD FACS SORP Aria-IIu machine. For intracellular staining of Gata3, staining of cell surface markers were followed by fixing and permeabilizing cells with Cytofix/cytoperm fixation/permeabilization kit (BD Pharmingen). Anti-Gata3-pacific blue (Biolegend) was then added according to the manufacturer's instructions (BD Biosciences-PharMingen). FACS was performed using the LSR–Fortessa machine (BD Pharmingen). Data analysis was performed using Kaluza software (Beckman Coulter). Absolute numbers of T and B cells were calculated using the total cell number multiplied by the percentage of T and B cells expressing specific markers divided by 100.

### BrdU labeling assay

Mice were intraperitoneally injected with BrdU (50 mg/kg) in sterile phosphate-buffered saline, sacrificed 14 hours post-injection, and single cell suspensions were harvested from the BM, spleen, thymus, and lymphoma. Two million cells were stained with cell surface antigens for 30 minutes on ice, and washed twice. The pelleted cells were resuspended in Fixation/Permeabilization solution (Cytofix/cytoperm fixation/permeabilization kit, BD Pharmingen) and incubated for 20 minutes on ice. After washing, the pelleted cells were resuspended in Perm/Wash buffer with addition of pacific blue conjugated anti-BrdU antibody (Invitrogen). The cells were incubated for 1 hour at room temperature in the dark and then analyzed by FACS. BrdU and B220 doubly positive BM cells, splenocytes, or lymphoma cells as well as BrdU positive thymocytes were determined. Gating criteria for determination of BrdU^+^ population in cells from the BM and thymus are shown in Supplementary Figure S2.

### Western blot and Q-RT-PCR

Tissue and cell lysates were prepared as previously reported [53]. Antibodies to Gata3 (HG3-31, Santa Cruz), and Gapdh (Ambion) were purchased commercially. Total RNA was extracted using the RNeasy kit (Qiagen) according to the manufacturer's protocol and cDNA was generated using the Omniscript RT Kit (Qiagen). QRT-PCR was performed as reported [53].

### LOH and IgH rearrangement analysis

For LOH analysis, genomic DNA was extracted from microdissected lymphomas or ear tissues in *p18*^−/−^;*Gata3*^+/−^ mice were analyzed with PCR as we previously described [54]. Primers used for detection of *Gata3* WT and knockout alleles were as follows: 5′- CCCCTTT CCCGGCTCTATCTT-3′ and 5′- GGGCCGGTTCTGC CCATT -3′; 5′-GGCATTCTCGCACGCTTCAAA-3′ and 5′-GGATGGGCACCACCCCGGTGAA -3′, respectively. DJ rearrangements of immunoglobulin heavy chain for DNA extracted from microdissected lymphomas were determined by PCR as previously described. DFS: 5′-AGGGATCCTTGTGAAGGGATCTACTACTGTG-3′ and JH4-C: 5′-AAAGACCTGCAGAGGCCATTCTTACC-3′.

### Lymphoma reconstitution

2 × 10^6^ primary cells isolated from a *p18*^−/−^;*Gata3*^+/−^ splenic lymphoma (immature B-cell lymphoma) or an asymptomatic spleen were injected into female NSG mice (6–8 weeks old) via tail vein (*n* = 3 for each group). Recipient mice were monitored daily and sacrificed four weeks post transplantation. Tissues and cells were then collected for histopathological and flow cytometric analyses.

### Statistical analysis

All data are presented as the mean ± SD for at least three individual experiments for each group. Quantitative results were analyzed by two-tailed Student's *t-test*. *P* < 0.05 was considered statistically significant. * represents *P* < 0.05; **, *P* < 0.01; and ***, *P* < 0.001.

## SUPPLEMENTARY MATERIALS FIGURES AND TABLES


