# Supplementary material for: Gata3 restrains B cell proliferation and cooperates with p18INK4c to repress B cell lymphomagenesis
Source: Oncotarget. 2016 Aug 31;7(39):64007–20. doi: 10.18632/oncotarget.11746 (PMC5325421; doi:10.18632/oncotarget.11746)
Supplement: Supplementary file 1 [file oncotarget-07-64007-s001.pdf]

Gata3 restrains B cell proliferation and cooperates with p18^{INK4c} to repress B cell lymphomagenesis

Supplementary Materials

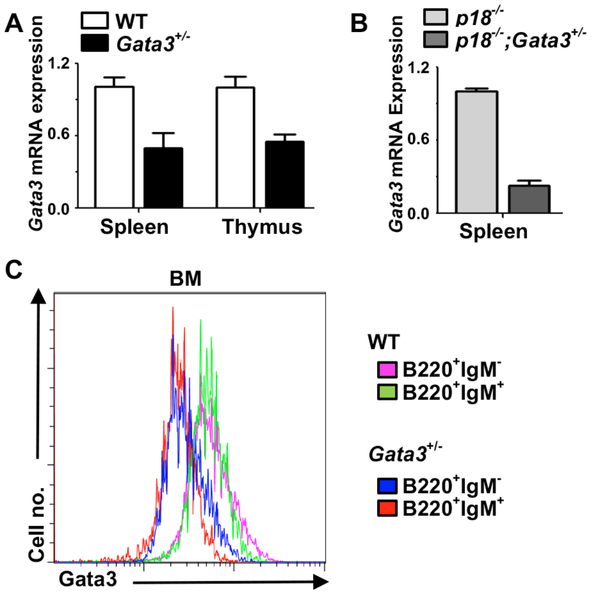

Supplementary Figure S1: (A) Cells from the spleen and thymus of WT and *Gata3*$^{+/-}$ mice at 2 months of age were analyzed for *Gata3* expression by Q-RT-PCR. (**B**). Cells from the spleen of *p18*$^{-/-}$ and *p18*$^{-/-}$;*Gata3*$^{+/-}$ mice at 2 months of age were analyzed for *Gata3* expression by Q-RT-PCR. Data represent the mean ± SD from triplicates of 2 mice per genotype. (**C**). B220^{+}IgM^{-} and B220^{+}IgM^{+} B cells from BM of WT and *Gata3*$^{+/-}$ mice at 2 months of age were analyzed for Gata3 expression by flow cytometry.

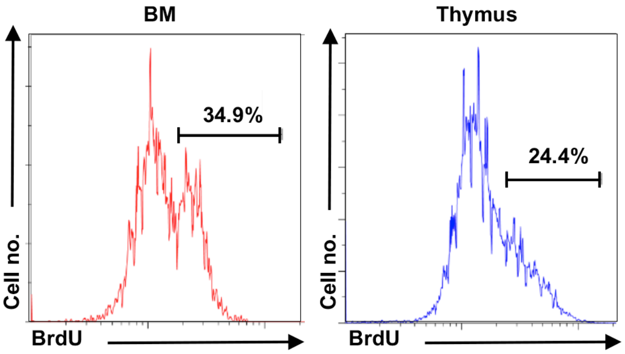

Supplementary Figure S2: Gating criteria for determination of BrdU$^+$ population in cells from BM and thymus. Cells from a mouse that had been injected with BrdU for 14 hours were stained with anti-BrdU antibody. Percentages of BrdU positive cells were indicated.

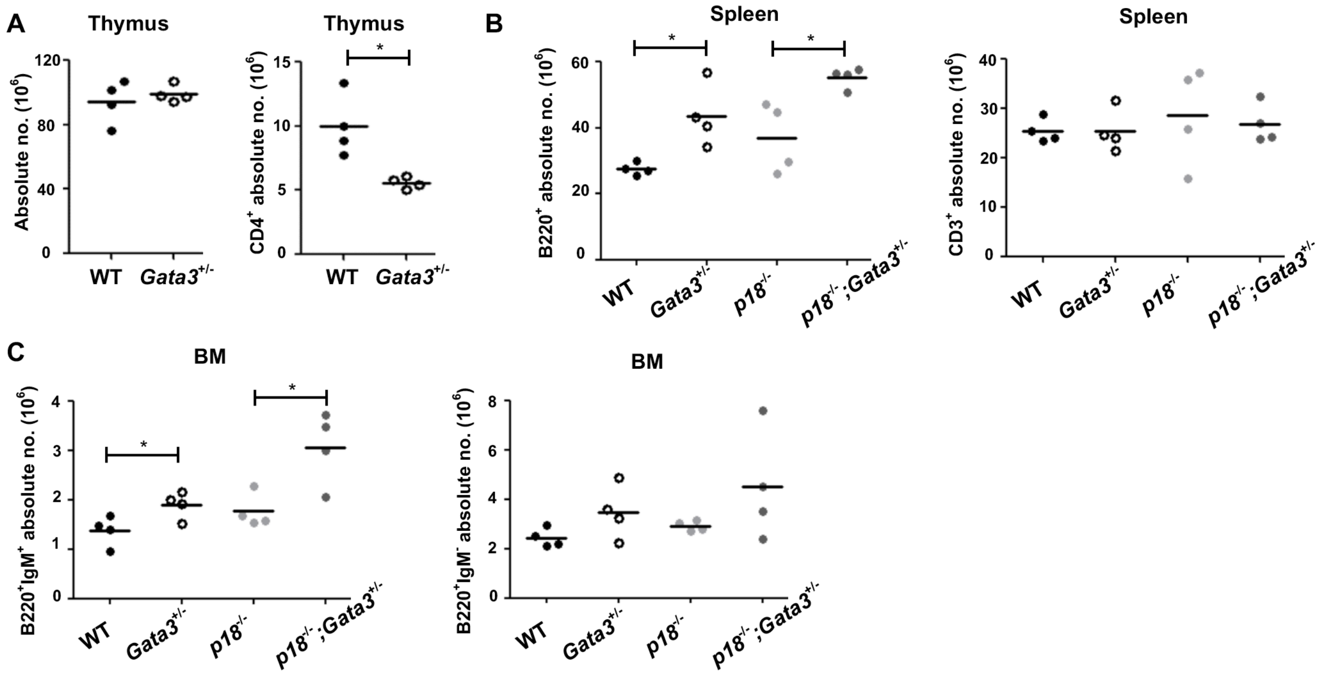

Supplementary Figure S3: Absolute T and B cell numbers in mice of different genotypes. (**A**) The absolute cell number of total thymocytes and CD4$^+$ thymocytes in WT and *Gata3*$^{+/-}$ mice at 2 months of age (n = 4) that was determined and calculated from the FACS profile. (**B**) The absolute cell number of B220$^+$ and CD3$^+$ cells in WT, *Gata3*$^{+/-}$, *p18*$^{-/-}$ and *p18*$^{-/-}$;*Gata3*$^{+/-}$ spleens that was determined and calculated from FACS profile. (**C**) The absolute cell number of B220$^+$IgM$^+$ and B220$^+$IgM$^-$ cells from WT, *Gata3*$^{+/-}$, *p18*$^{-/-}$ and *p18*$^{-/-}$; *Gata3*$^{+/-}$ BM that was determined and calculated from the FACS profile. *$P < 0.05$.

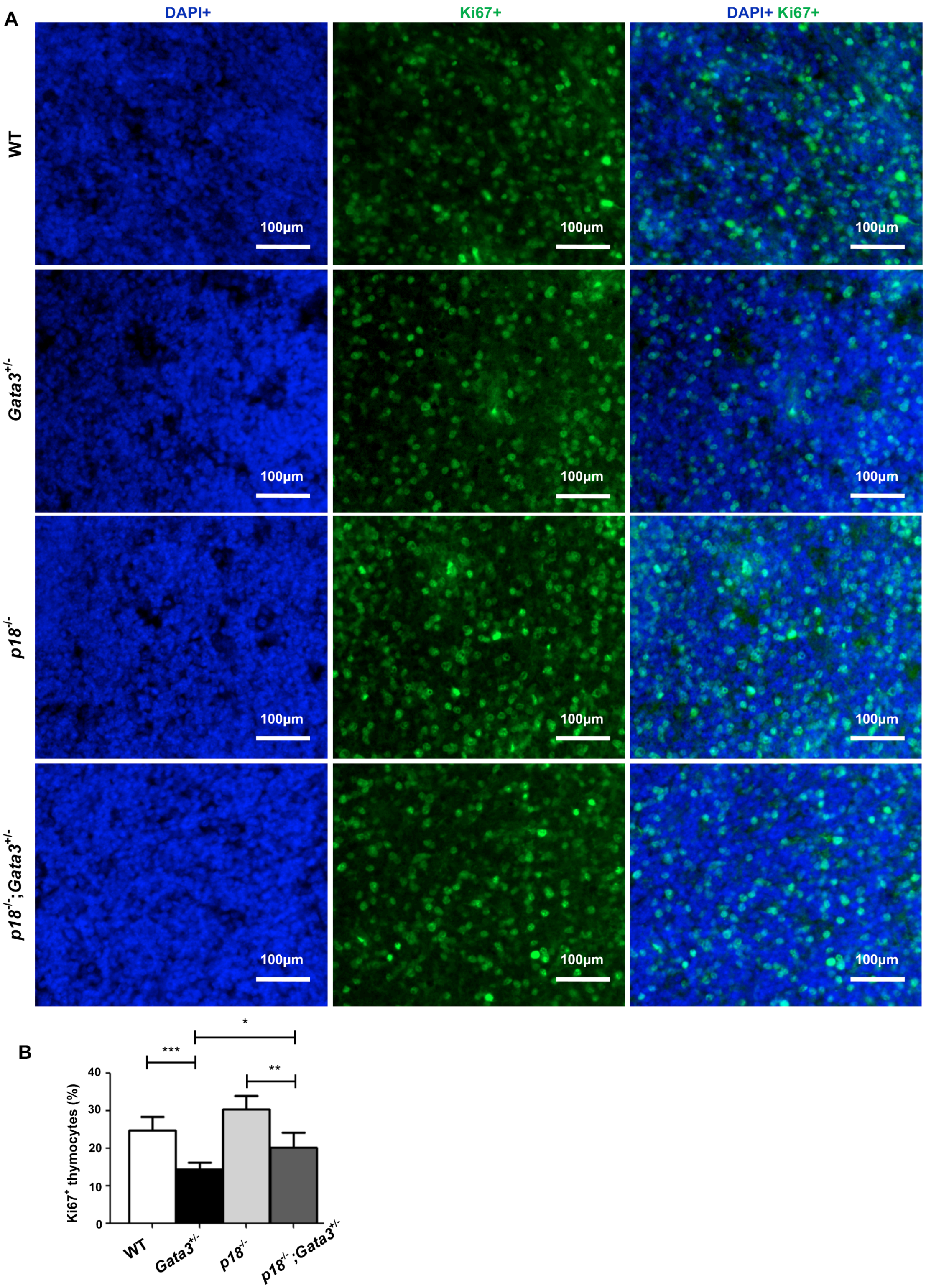

Supplementary Figure S4: (**A**) Immunofluorescence staining of Ki67 (green, nuclear staining) and DAPI (Blue, nuclear staining) in thymus from WT, Gata3$^{+/-}$, *p18*$^{-/-}$ and *p18*$^{-/-}$;*Gata3*$^{+/-}$ mice. (**B**). Quantification of Ki67 positive cells. 5 random fields in at least two cut sections from each thymus were examined and at least 500 DAPI positive cells per field were counted (more than 2500 cells per thymus). The average percentages of Ki67 positive cells were calculated from WT, *Gata3*$^{+/-}$, *p18*$^{-/-}$ and *p18*$^{-/-}$;*Gata3*$^{+/-}$ mice and the results represent the mean ± SD of two animals per group.

Supplementary Table S1: Summary of the B and T cell populations (%) in mutant mice

Genotype	WT		*Gata3*$^{+/-}$		*p18*$^{-/-}$		*p18*$^{-/-}$;*Gata3*$^{+/-}$	
**Spleen**	**CD3^{+}**	**B220^{+}**	**CD3^{+}**	**B220^{+}**	**CD3^{+}**	**B220^{+}**	**CD3^{+}**	**B220^{+}**
Experiment 1	40.2	45.3	24.4	64.6	39.9	50.6	29.1	61.8
Experiment 1	46.2	46.5	39.3	52.7	40.0	50.0	29.5	60.8
Experiment 1	47.5	45.5	40.5	51.7	43.6	50.2	33.6	59.7
Experiment 1	41.0	52.6	24.3	63.5	34.0	56.8	27.3	64.4
Experiment 1	39.0	50.0	32.0	55.8	33.0	57.3	26.1	64.7
Mean ± SD	42.8 ± 3.8	48.0 ± 3.2	32.0 ± 7.7	57.7 ± 6.0	38.1 ± 4.4	52.9 ± 3.7	29.1 ± 2.9	62.3 ± 2.2
**BM**	**B220^{+} IgM^{+}**	**B220^{+} IgM^{-}**	**B220^{+} IgM^{+}**	**B220^{+} IgM^{-}**	**B220^{+} IgM^{+}**	**B220^{+} IgM^{-}**	**B220^{+} IgM^{+}**	**B220^{+} IgM^{-}**
Experiment 1	8.87	10.50	12.93	15.27	10.9	17.36	13.99	21.19
Experiment 2	6.89	15.74	10.8	22.85	8.79	15.06	15.40	15.55
Experiment 3	9.44	16.40	10.3	24.47	10.29	17.66	13.46	15.69
Experiment 4	8.4	13.6	10.9	15.0	9.42	15.34	16.00	17.20
Experiment 5	9.2	17.4	12.3	22.3	12.90	22.2	14.4	29.4
Mean ± SD	8.6 ± 1.0	14.7 ± 2.8	11.4 ± 1.1	20.0 ± 4.5	10.5 ± 1.8	17.5 ± 2.8	14.7 ± 1.0	19.8 ± 5.8
**Thymus**	**CD4^{+}**		**CD4^{+}**		**CD4^{+}**		**CD4^{+}**	
Experiment 1	18.0		13.0		13.95		9.67	
Experiment 2	12.13		8.86		9.11		8.77	
Experiment 3	14.18		7.99		9.75		7.02	
Experiment 4	16.79		7.50		14.25		10.14	
Experiment 5	11.7		8.2		12.0		7.6	
Mean ± SD	14.6 ± 2.8		9.1 ± 2.2		11.8 ± 2.3		8.6 ± 1.3	

Supplementary Table S2: Summary of the BrdU incorporation (%) in thymocytes and B cells in the bone marrow

Genotype	WT	*Gata3*$^{+/-}$	*p18*$^{-/-}$	*p18*$^{-/-}$;*Gata3*$^{+/-}$
**BM**	**B220^{+}Brdu^{+}**	**B220^{+}Brdu^{+}**	**B220^{+}Brdu^{+}**	**B220^{+}Brdu^{+}**
Experiment 1	7.13	12.85	14.57	24.32
Experiment 2	8.7	17.5	14.7	33.5
Experiment 3	8.3	11.9	15.08	24.8
Mean ± SD	8.0 ± 0.8	14.1 ± 2.9	14.8 ± 0.3	27.5 ± 5.1
**Thymus**	**Thymocytes**	**Thymocytes**	**Thymocytes**	**Thymocytes**
Experiment 1	17.2	9.6	21.9	13.9
Experiment 2	24.4	6.88	22.1	16.8
Experiment 3	21.1	7.9	22.0	17.6
Mean ± SD	20.9 ± 3.6	8.1 ± 1.3	22.0 ± 0.1	16.1 ± 1.9
